# The Prognostic Implications of Synchronous Cancers in Breast Cancer Patients

**DOI:** 10.7759/cureus.68380

**Published:** 2024-09-01

**Authors:** Alexandru Oprita, Horia Cotan, Cornelia Nitipir

**Affiliations:** 1 Oncology, Carol Davila University of Medicine and Pharmacy, Bucharest, ROU; 2 Oncology, Saint Nicholas Hospital, Pitesti, ROU; 3 Oncology, Elias Emergency University Hospital, Bucharest, ROU

**Keywords:** prognostic factor, synchronous bilateral breast cancer, synchronous cancer, synchronous carcinoma, synchronous primary cancers

## Abstract

Objective: This study aims to examine the prognostic value of synchronous cancer diagnosis following an initial diagnosis of breast cancer, with a focus on site-specific survival rates and the correlation between primary breast cancer and secondary cancers.

Methods: We conducted a retrospective analysis of patients treated at Saint Nicholas Hospital in Pitesti, Romania, from January 2016 to January 2024. The inclusion criteria were a confirmed diagnosis of primary breast cancer and a secondary synchronous cancer diagnosed within two months. Data collection included demographic, clinical, and pathological characteristics, as well as treatment details and follow-up outcomes. Statistical analyses were performed using SPSS software version 26.0 (IBM Corp., Armonk, New York, USA), employing Kaplan-Meier survival curves, Cox regression models, and other relevant statistical tests.

Results: Out of 73 initially identified patients, 49 met the inclusion criteria. The mean age was 59.6 years, with most patients being postmenopausal. Synchronous cancers were primarily contralateral breast cancer (44.9%) and female genital organ cancer (12.24%). Patients with synchronous bilateral breast cancer had significantly better overall survival (33 months) compared to those with other synchronous cancers (23.5 months). Multivariate analysis indicated that synchronous non-breast cancers were associated with a higher risk of death (hazard ratio (HR)=1.6, 95% CI: 1.22-2.10, p=0.003).

Conclusion: Synchronous cancer diagnosis following an initial breast cancer diagnosis significantly impacts prognosis, with synchronous bilateral breast cancer associated with better survival outcomes compared to other synchronous cancers. These findings underscore the importance of vigilant screening and personalized treatment strategies for patients with synchronous malignancies.

## Introduction

Data released by various cancer societies indicate a consistent decline in cancer mortality rates over the three decades [1]. This notable progress is attributed to advancements in treatments and earlier detection methods, underscoring the growing significance of potential long-term consequences. Second cancer occurrences now make up 15% to 20% of all cancer diagnoses recorded in cancer registries [2]. Among pediatric patients, the risk of developing a second cancer is notably higher compared to the general population, ranging from three- to 10-fold higher. Regarding solid cancers, standardized incidence ratios (SIRs), representing the ratio of observed to expected numbers of second cancers, decline with increasing age at the time of childhood cancer diagnosis and with longer follow-up times, whereas excess absolute risk (EAR), defined as the difference between observed and expected numbers of second cancers, increases with longer follow-up periods. Conversely, for hematologic malignancies, SIR and EAR peak between five- and nine-years post-diagnosis before declining.

The probability of a synchronous cancer diagnosis following an initial breast cancer diagnosis is an important topic in oncology. Synchronous cancers refer to multiple primary tumors diagnosed simultaneously or within a short period.

A significant study found that among patients with multiple primary cancers, a notable portion had synchronous cancers, with breast cancer being the most common primary cancer leading to synchronous secondary cancers ​[3]. Specifically, breast cancer patients have been reported to have a 34.6% incidence of developing synchronous cancers, which emphasizes the importance of vigilant screening and monitoring​ [3]​.

Synchronous bilateral breast cancer (SBBC) is relatively rare but crucial for patient outcomes. Studies indicate that the prognosis for SBBC can be similar to unilateral breast cancer if treated appropriately, often involving breast-conserving surgery. This underscores the necessity of screening the contralateral breast in patients diagnosed with unilateral breast cancer to detect potential synchronous tumors early​ [4]​.

Additional research highlights that synchronous cancers often involve organs such as the ovaries, liver, and colorectal region, indicating a broad spectrum of secondary cancer risks following a primary breast cancer diagnosis​ [5]​.

The purpose of this study is to determine site-specific survival rates for patients diagnosed with primary breast cancer and synchronous secondary cancer. Our goal is to identify the correlation between primary breast cancer and other secondary cancers. Synchronous multiple primary cancers were classified using a cutoff of two months, following the surveillance, epidemiology, and end results (SEER) criteria [6,7]. Essentially, cancers diagnosed within two months of each other are considered synchronous.

## Materials and methods

Study population

All patients involved in this retrospective study received treatment and follow-up at the Oncology Department at Saint Nicholas Hospital in Pitesti, Romania, from January 2016 to January 2024. All participants in this study were diagnosed with primary breast cancer. Treatment for these patients primarily involved hormone therapy, targeted therapy, or chemotherapy based on the immunophenotypic profile of the tumor. All patients included in this study were also diagnosed with a secondary synchronous cancer that involved the contralateral breast or another organ.

The inclusion criteria required a confirmed positive diagnosis of breast cancer (BC) through histopathological and immunohistochemical assessments, along with precise clinical staging. Pathological staging was conducted by a seasoned pathologist utilizing the American Joint Committee on Cancer (AJCC) tumor, node, and metastasis (TNM) Staging Classification for Breast Cancer 8th edition, 2017. Clinical staging involved comprehensive computed tomography (CT) or magnetic resonance imaging (MRI) scans of the chest, abdomen, and pelvis, along with brain MRI scans for symptomatic patients. Lesions suspected to be metastases but not definitively identified as such on imaging underwent biopsy for histopathological and immunohistochemical confirmation. Secondary synchronous cancers were staged according to the proper TNM classification. All secondary cancers were also confirmed through histopathological and immunohistochemical assessment.

The following exclusion criteria were established: Patients younger than 18 years at the time of breast cancer diagnosis were excluded to focus on the adult population. Those without a confirmed diagnosis of primary breast cancer or with a prior history of any cancer were also excluded. To maintain a consistent timeframe, patients whose initial breast cancer diagnosis fell outside the predefined study period were not considered. Additionally, patients lacking sufficient follow-up data to confirm secondary synchronous cancers, those with secondary cancers diagnosed more than two months after the initial breast cancer (following SEER criteria), and those with hematologic malignancies as secondary cancers were excluded. Incomplete or missing critical medical records, non-histologically confirmed cancers, participation in experimental treatment trials, and secondary cancers diagnosed post-mortem all led to exclusion in order to ensure data integrity and diagnostic accuracy.

**Figure 1:**
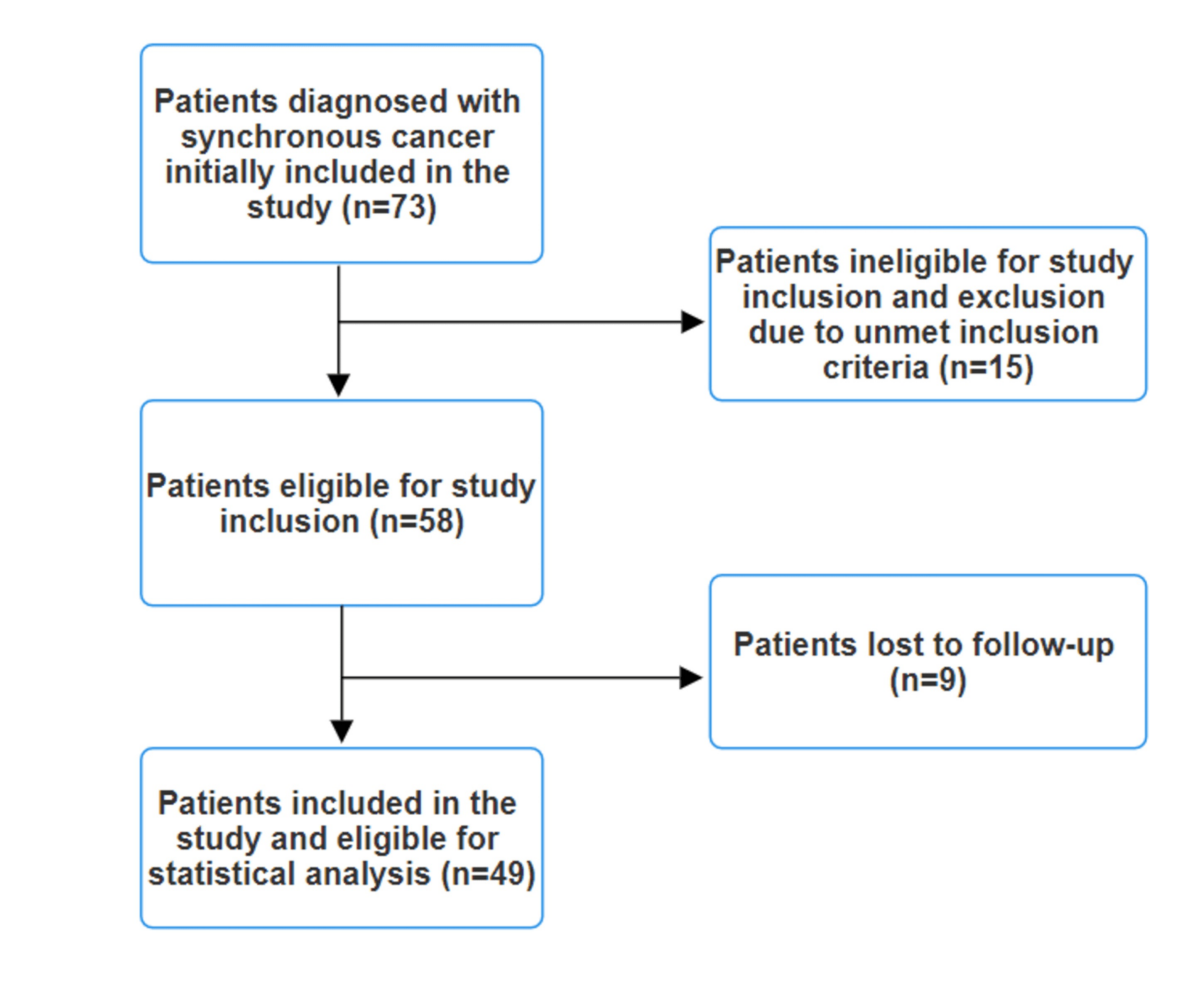
Study flow chart.

Initially, 73 patients were included in the study. However, after applying the exclusion criteria, a total of 49 patients remained eligible for inclusion in the study (Figure 1).

Data collection

Data collection involved gathering comprehensive information from patient records sourced from oncology departments, cancer registries, and electronic health records from participating hospitals and clinics. We included patients with a confirmed primary breast cancer diagnosis within the study period while excluding those who met predefined exclusion criteria. Demographic data, such as age, sex, race, ethnicity, and family cancer history, were extracted from medical records. Clinical and pathological data encompassed details of the primary breast cancer, including diagnosis date, tumor characteristics (size, grade, subtype, hormone receptor status, stage), and treatment specifics (surgery, chemotherapy, radiation, hormone, and targeted therapy). For secondary synchronous cancers, we documented the diagnosis date, tumor characteristics, and treatment details. Follow-up data will track overall survival (OS), progression-free survival (PFS), recurrence, metastasis, complications, and side effects. Laboratory and imaging data included relevant blood tests, biomarker levels, genetic test results, and imaging reports. Ensuring data quality involves cross-checking entries, data cleaning, and maintaining inter-rater reliability.

Statistical analysis

Statistical analyses were conducted using SPSS software version 26.0 (IBM Corp., Armonk, New York, USA). Descriptive statistics were used to report patient and disease characteristics. Differences between groups were analyzed using Pearson's Chi-squared test or Fisher's exact test for categorical variables, and the two-sample T-test or Wilcoxon rank-sum test for continuous variables. The Kaplan-Meier method was utilized to evaluate median survival time, with the log-rank test used to compare survival distributions between groups, highlighting significant differences in survival outcomes. Univariate and multivariate Cox regression models were employed to assess the relationship between synchronous breast cancer and the risk of death or recurrence, adjusting for potential confounders. Overall survival was calculated from the diagnosis date to the date of death, and progression-free survival from the initiation of treatment to the first documented progression according to Response Evaluation Criteria in Solid Tumors (RECIST) criteria. The overall response rate (ORR) was defined as the proportion of patients achieving either a partial or complete response to the first line of therapy. Partial response, complete response, and stable disease were categorized as favorable responses, while progressive disease was considered unfavorable. Continuous variables with normal distribution were expressed as mean and standard deviation (SD), whereas those without normal distribution were presented as median and quartiles. Categorical variables were reported as counts (n) and percentages (%). A p-value of less than 0.05 was considered statistically significant.

## Results

Patient’s characteristics

Out of 73 cases of synchronous cancer patients treated only 49 were included in this analysis after the application of inclusion/exclusion criteria. The patients were all female and ranged in age from 34 to 83 years, with an average age of 59.6 years. Most patients (n=36; 73.5%) were breast cancer gene (BRCA) 1/2 wild-type, while only 13 (26.5%) patients were BRCA 1/2 mutant. Treatment received varied depending on immunophenotype and clinical stage of the primary tumor; most patients (n=34; 69.4%) received hormone therapy and cyclin-dependent kinases 4 and 6 (CDK4/6) inhibitors if indicated, while 15 (30.6%) received chemotherapy-associated with anti-human epidermal growth factor receptor 2 (HER2) therapy or immunotherapy depending on the immunophenotype of the tumor. When we classified the primary breast cancer by immunophenotype, the majority of patients (n=22, 44%) were Luminal A, while 12 (24%) were Luminal B HER2 negative, 3 (6%) Luminal B HER2 positive, 6 (12%) hormone receptor-negative HER2 positive and 6 (12%) triple-negative breast cancer (TNBC). This data is shown in Table 1.

**Table 1:** Baseline clinical characteristics of patients. Breast cancer molecular subtypes: Luminal A: ER+ and/or PR+ (PR ≥ 20%), HER2-, Ki-67 < 14%; Luminal B (HER2-): ER+ and/or PR+ (PR < 20%), HER2-, Ki-67 ≥ 14%; Luminal B (HER2+): ER+ and/or PR+, HER2+, Ki-67 any; HER2-enriched: ER−, PR−, HER2+; triple-negative breast cancer (TNBC): ER−, PR−, HER2-; BRCA: breast cancer gene; HT: hormone therapy; CHT: chemotherapy, TNM: tumor, node, and metastasis; HER2: human epidermal growth factor receptor 2.

	Synchronous breast cancer (n=18)	Other synchronous cancers (n=31)
Age, years, mean (SD)	60.6 ± 7.13	58.6 ± 4.31
Primary breast cancer type, n (%)		
Ductal	13 (72.2 %)	24 (77.4 %)
Lobular	5 (27.8 %)	7 (22.6 %)
Primary breast cancer immunophenotype, n (%)		
Luminal A	9 (50 %)	13 (41.9 %)
Luminal B HER2−	3 (16.7 %)	9 (29 %)
Luminal B HER2+	2 (11.1%)	1 (3.2 %)
HR negative HER2+	2 (11.1%)	4 (12.9 %)
TNBC	2 (11.1%)	4 (12.9 %)
Menopausal status, n (%)		
Menopause	14 (77.8 %)	25 (80.6%)
Premenopause	4 (22.2 %)	6 (19.4%)
BRCA 1/2 mutational status, n (%)		
Wild-type	14 (77.8 %)	22 (71 %)
Mutant	4 (22.2 %)	9 (29 %)
Primary breast cancer T-stage		
T1	1 (5.6 %)	0 (0 %)
T2	5 (27.8 %)	13 (41.9 %)
T3	7 (38.9 %)	13 (41.9 %)
T4	5 (27.8 %)	5 (16.1%)
Primary breast cancer N stage		
N0	5 (27.8 %)	8 (25.8 %)
N1	4 (22.2 %)	10 (32.3 %)
N2	9 (50 %)	9 (29 %)
N3	0 (0%)	4 (12.9 %)
TNM stage		
Stage I	3 (16.7 %)	5 (16.1 %)
Stage II	1 (5.6%)	4 (12.9 %)
Stage III	4 (22.2 %)	10 (32.3 %)
Stage IV	10 (55.6%)	12 (38.7 %)
Treatment		
HT	13 (72.2 %)	21 (67.7 %)
CHT	5 (27.8 %)	10 (32.3 %)

**Figure 2:**
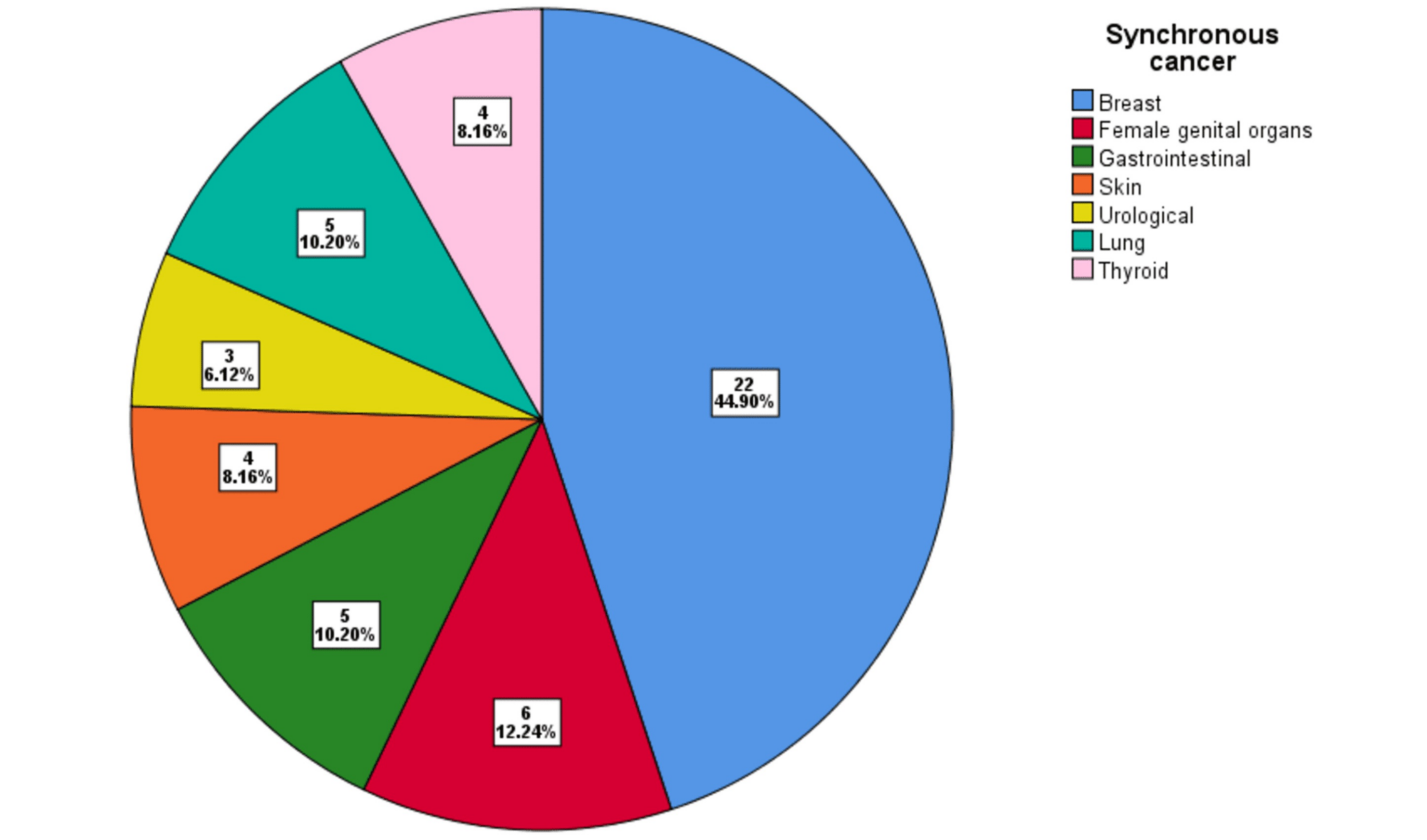
Pie chart illustrating the distribution of various types of synchronous cancers.

When patients were categorized based on the presence of synchronous cancer, the majority (n=18, 44.9%) had synchronous breast cancer. Additionally, six patients (12.2%) had synchronous genital organ cancer, 5 (10.2%) had gastrointestinal cancer, 4 (8.2%) had skin cancer, 3 (6.1%) had cancer, 5 (10.2%) had pulmonary cancer, and 4 (8.2%) had thyroid cancer (Figure 2).

Clinical outcome stratified by synchronous cancer

**Figure 3:**
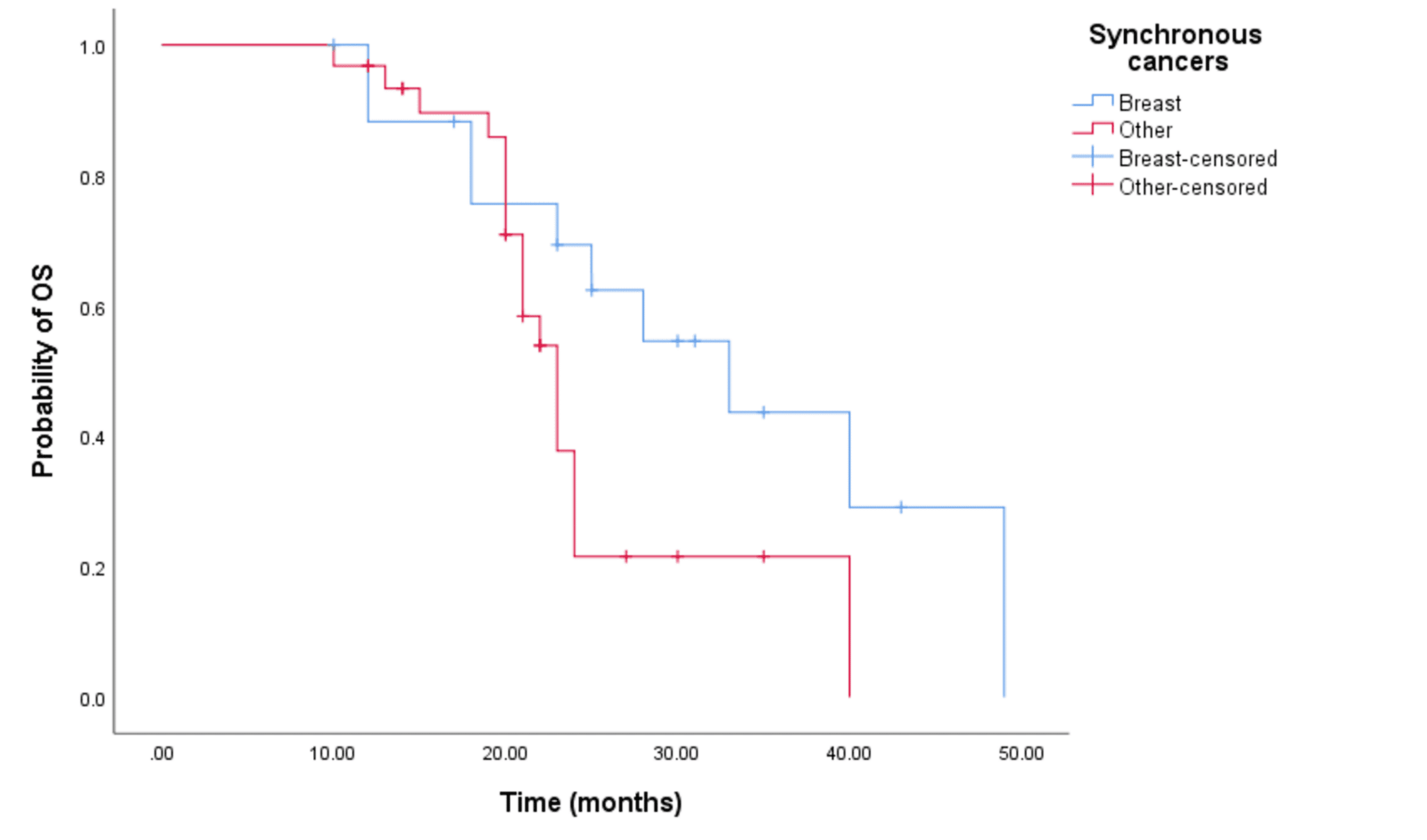
Comparative analysis of Kaplan–Meier curves between synchronous breast cancer patients and other synchronous cancer patients for overall survival (OS).

**Figure 4:**
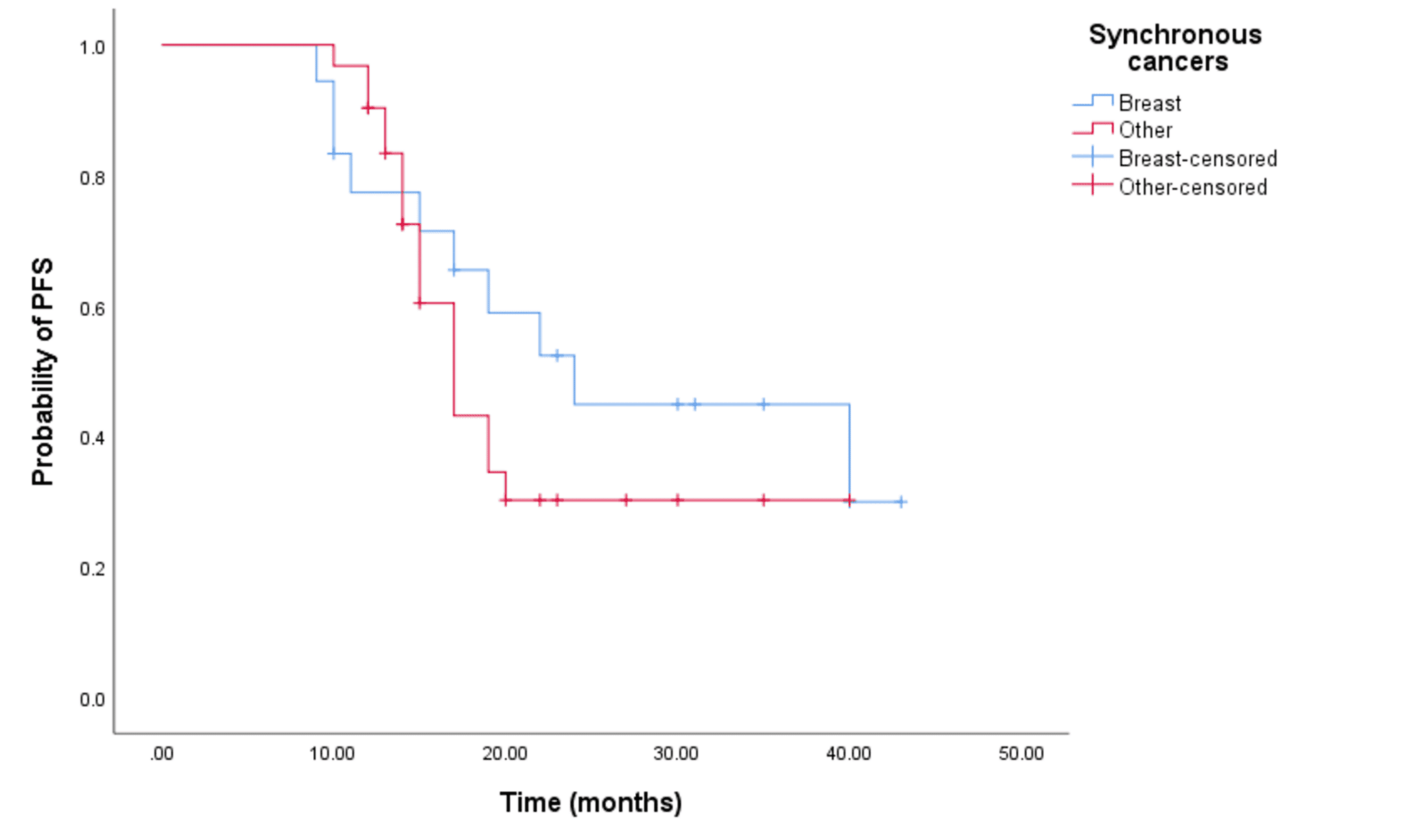
Comparative analysis of Kaplan–Meier curves between synchronous breast cancer patients and other synchronous cancer patients for progression-free survival (PFS).

For survival analysis, patients that have synchronous breast cancer have been classified in one group (bilateral breast cancer), and patients with other synchronous cancers in another group. The median overall survival (MOS) and progression-free survival (PFS) were 24 months (95% CI: 22.445-25.555) and 19 months (95% CI: 16.019-21.981), respectively. There was a statistically significant in OS time (p=0.05) between groups, with OS for patients with bilateral breast cancer being 33 months (95% CI: 21.072-44.928) and 23.5 months (95% CI: 21.557-24.443) for those other synchronous cancers. Although not statistically significant, there was a numerical difference in PFS between groups. The longest progression-free survival was observed in patients with bilateral breast cancer, with a PFS of 24 months (95% CI: 15.163-32.837, p=0.394) compared to patients with other synchronous cancers which have a PFS of 17 months (95% CI: 14.747-19.253). This data is represented by the Kaplan-Meier curves below (Figures 3, 4).

Multivariate analysis of the entire cohort

The prognostic value of synchronous cancer has been confirmed in both the bilateral breast cancer cohort and other synchronous cancer cohorts through multivariate analysis (Cox regression). The hazard ratio (HR) for death among patients with synchronous breast-non-breast cancer was 1.6 (95% CI: 1.22-2.10, p=0.003, Table 2), while the HR for cancer progression was 1.15 (95% CI: 1.05-2.00, p=0.345, Table 2).

**Table 2:** Multivariate Cox regression analyses to identify predictors for increased risk of death and tumor progression, considering synchronous cancer as an independent variable. BRCA: breast cancer gene; TNBC: triple-negative breast cancer; HER2: human epidermal growth factor receptor 2; BRCA: breast cancer gene; OS: overall survival; PFS: progression-free survival; HR: hazard ratio.

Test variables	OS HR (95% CI)	p-value	PFS HR (95% CI)	p-value
Synchronous breast cancer (ref.)/other synchronous cancers	1.60 (1.22-2.10)	0.003	1.15 (1.05-2.00)	0.345
BRCA 1/2 mutational status wild-type (ref.)/mutant	1.03 (0.87-1.23)	0.128	1.173 (1.04-1.25)	0.383
Ductal (ref.)/lobular	1.25 (1.10-1.42)	0.288	1.30 (1.10-1.55)	0.087
Stage I-III (ref.)/stage IV	1.35 (1.15-1.58)	0.005	1.62 (1.22-2.23)	0.004
Luminal subtype (ref.)/HER2 enriched	1.50 (1.30-1.73)	<0.001	1.72 (1.40-2.09)	0.003
Luminal subtype (ref.)/TNBC	1.40 (1.12-1.75)	0.006	1.43 (1.10-2.20)	0.008

## Discussion

Examining the prognostic value of synchronous cancer diagnosis following an initial diagnosis of breast cancer presents significant clinical implications. The occurrence of multiple primary malignancies in a single patient, especially when one of the cancers is breast cancer, can complicate treatment decisions and impact overall survival outcomes. Previous studies have indicated that synchronous cancers, although relatively rare, pose distinct challenges and require nuanced treatment strategies compared to single primary cancers [8,9].

In our study, the focus was to investigate the survival rates and clinical outcomes of patients diagnosed with both primary breast cancer and synchronous secondary cancers. The classification of synchronous cancers, defined as those occurring within two months of each other, adheres to the SEER criteria. This stringent timeline ensures that the cancers are indeed synchronous rather than metachronous, which typically emerge after a longer interval following the initial cancer diagnosis. Patients with a BRCA mutation are known to have an increased risk of developing multiple primary malignancies​ [10]​. Additionally, the presence of synchronous cancers in organs such as the gastrointestinal tract, urological system, and thyroid has been documented, underscoring the need for comprehensive screening and monitoring in patients diagnosed with primary breast cancer​ [11]. Despite these studies, our study did not find any significant difference between BRCA 1/2 status in synchronous breast cancer or other synchronous cancers. The integration of genetic profiling and biomarkers, such as BRCA status and hormone receptor status, is crucial in tailoring personalized treatment plans for patients with synchronous cancers. Studies have shown that the genomic landscape of breast cancer can influence the development and progression of secondary cancers, thereby affecting prognosis and therapeutic outcomes​ [4]​.

Our observation revealed that the most common synchronous neoplasms were contralateral breast cancer (44.9%) and genital organ cancer (12.24%). According to some literature, a lobular component of breast carcinoma nearly doubles the risk of developing contralateral breast cancer, particularly synchronous bilateral breast cancer [12,13]. In our study, 27% of patients with bilateral synchronous breast cancer had lobular histology.

Published data indicate that age and menopausal status at breast cancer diagnosis are risk factors for the development of a second cancer [12,14]. Lv et al. reported that 84.6% of patients with synchronous malignancies were over 50 years old [14]. In our cohort, the average age at diagnosis was 59.6 years, with the majority being postmenopausal. The occurrence of a second primary is associated with a significantly increased risk of death and significantly worse survival [11]. Results published by Carmichael et al. showed that significantly worse overall survival is observed among patients with synchronous contralateral breast cancer in comparison to metachronous or unilateral breast cancer [15]. A retrospective study [16] analyzing the impact of synchronous breast cancer on survival compared to other synchronous cancers reported that synchronous breast-breast cancer was associated with superior survival compared to synchronous breast-non-breast cancer. Similar findings were reported in our study, with a significant survival advantage among patients with synchronous breast-breast cancer compared to other cancers.

The study's retrospective design may introduce selection bias and limit the ability to establish causal relationships between synchronous cancer diagnosis and breast cancer prognosis. Additionally, the sample size may be limited, reducing the generalizability of the findings to broader populations. Larger, multi-center studies are needed to validate the results. Reliance on medical records and patient self-reports for data collection may result in inaccuracies or incomplete data, potentially affecting the study's outcomes. The length of follow-up may vary among patients, potentially leading to an underestimation or overestimation of the true prognostic value of synchronous cancer diagnosis.

There may be unmeasured confounding variables, such as genetic predispositions or environmental factors, that could influence the prognosis of patients with synchronous cancers. Differences in treatment protocols for both breast cancer and synchronous cancers across institutions or over time may impact patient outcomes and limit the study’s ability to standardize findings. The diverse nature of synchronous cancers (e.g., different types and stages) may complicate the analysis and interpretation of their prognostic value in relation to breast cancer.

The absence of a well-matched control group of breast cancer patients without synchronous cancers may limit the ability to isolate the impact of synchronous cancer on prognosis. Patients who survive long enough to develop a second cancer may inherently have different characteristics compared to those who do not, introducing survivorship bias. Finally, the study may not adequately account for the influence of socioeconomic and demographic factors on patient outcomes, which could confound the results.

Our study had a follow-up period of up to 42 months with a median follow-up of 31 months. However, there are some limitations to consider. The relatively short follow-up period may not fully capture long-term outcomes or late recurrences, which are crucial in breast cancer prognosis. Additionally, the median follow-up of 31 months might limit the ability to observe the full impact of synchronous cancers on overall survival and disease progression. Therefore, longer follow-up studies are needed to better understand the long-term prognostic implications of synchronous cancers in these patients.

## Conclusions

Overall, the prognostic value of synchronous cancer diagnosis following an initial diagnosis of breast cancer emphasizes the need for ongoing research and clinical trials. Future studies should aim to validate these findings in larger, multi-center cohorts to enhance the generalizability and robustness of the data. Additionally, exploring the underlying biological mechanisms driving the development of synchronous cancers will provide deeper insights into potential therapeutic targets and intervention strategies.
