# Correction: The Prognostic Implications of Synchronous Cancers in Breast Cancer Patients

**DOI:** 10.7759/cureus.c285

**Published:** 2025-09-08

**Authors:** Alexandru Oprita, Horia Cotan, Cornelia Nitipir

**Affiliations:** 1 Oncology, Carol Davila University of Medicine and Pharmacy, Bucharest, ROU; 2 Oncology, Saint Nicholas Hospital, Pitesti, ROU; 3 Oncology, Elias Emergency University Hospital, Bucharest, ROU

This article has been revised to add the following affiliation for Alexandru Vlad Oprita: Oncology, Carol Davila University of Medicine and Pharmacy, Bucharest, Romania.

